# The Therapeutic Benefits of Outdoor Experiences in India

**DOI:** 10.3390/bs15091144

**Published:** 2025-08-22

**Authors:** Soumya J. Mitra, Vinathe Sharma-Brymer, Denise Mitten, Janet Ady

**Affiliations:** 1 Department of Parks, Recreation & Tourism, College of Health, University of Utah, Salt Lake City, UT 84112, USA; 2 School of Law & Society, University of the Sunshine Coast, Sippy Downs, QLD 4556, Australia; vsharmabrymer@usc.edu.au; 3 Adventure Education & Sustainability Education, Prescott College, Prescott, AZ 86301, USA; dmitten@prescott.edu; 4 Education for Sustainability, Prescott College, Prescott, AZ 86301, USA; jady@prescott.edu

**Keywords:** outdoor experiences, therapeutic outdoor practices, mental health, India, decolonial ecopsychology

## Abstract

Drawing on in-depth interviews and thematic analysis, this study explores the therapeutic benefits of outdoor experiences through the lived experiences of 24 outdoor practitioners, including educators, environmentalists, therapists, and program leaders. Three core themes emerged: (a) nature as an emotional regulator and reflective space; (b) therapeutic benefits of human–nature relationships; and (c) decolonial, bioregional, and cultural healing. Although practitioners facilitated physical challenges and skill-building for their participants, they primarily described outdoor experiences as relational, somatic, and culturally rooted practices that foster emotional regulation, grief processing, identity integration, and social inclusion. Healing emerged through solitude, silence, ancestral connections, sacred landscapes, inclusive dynamics, and the restoration of cultural knowledge. This study’s results challenge Western-centric outdoor education models by foregrounding Indigenous and postcolonial perspectives embedded in Indian ecological traditions. The results contribute to global discussions on decolonizing outdoor fields and offer implications for culturally responsive, emotionally safe, and ecologically grounded practices.

## 1. Introduction

Outdoor recreation and nature-based experiences are increasingly recognized for their role in promoting mental health and encouraging positive behavioral outcomes across the lifespan. A growing body of interdisciplinary research demonstrates that engagement with natural environments reduces stress, enhances mood, supports resilience, and fosters emotional regulation (Antonelli et al., 2019; Bowen et al., 2016; Bratman et al., 2019; Brymer et al., 2019; Ewert et al., 2021). Outdoor experiences, in particular, extend beyond physical challenges and skill acquisition, offering opportunities for healing, emotional restoration, social bonding, and self-discovery (Down et al., 2023; Ewert et al., 2021; Mitten, 2017).

Despite the growing acknowledgment of therapeutic outdoor experiences, dominant frameworks within outdoor fields often remain shaped by Anglo-Euro-American perspectives emphasizing structured facilitation, perception of risk, peak performance, and use of the natural environment for challenge and accomplishment (Mitten, 2017). These perspectives often overlook culturally embedded, relational, and somatic epistemologies prevalent across South Asian cultures, particularly within pre- and postcolonial contexts (Mitra et al., 2024). In India, structured outdoor programs evolved through hybrid lineages influenced initially by traditional ecological knowledge and spiritual practice, followed by colonial mountaineering traditions and later by international adventure models and contemporary ecological movements inviting youth into deeper and more intentional connection with land and ecosystems—yet, this complexity is underrepresented in global scholarship (Mitra, 2022).

Indian ecological and cultural traditions have long incorporated outdoor practices such as pilgrimage, communal ritual, and daily interactions with natural environments as avenues for mental, emotional, and social well-being (Sharma-Brymer, 2018, 2022). However, scholarly attention remains limited regarding how Indian practitioners conceptualize therapeutic dimensions of outdoor experiences or how traditional and contemporary healing practices integrate within their programs. This study addresses this gap by exploring the following question: In what ways do outdoor practitioners in India perceive and articulate the therapeutic dimensions of outdoor experiences? This includes supporting emotional, cultural, and relational healing, and how these are shaped by local ecological and spiritual traditions.

The research examines the question through in-depth interviews with 24 practitioners from India, working across education, tourism, conservation, and therapeutic settings. Practitioners’ narratives reveal how emotional regulation, cultural reconnection, and community well-being are fostered through everyday outdoor experiences. By highlighting Indigenous and decolonial perspectives, this work challenges dominant paradigms and contributes to a broader, more inclusive understanding of the therapeutic and behavioral potential of outdoor experiences.

### 1.1. Background

A growing body of research supports the claim that nature-based experiences positively influence mental health and emotional well-being (Ewert et al., 2021). Studies across disciplines, from psychology and public health to education and environmental science, have shown that positive relationships with, and exposure to, natural environments are associated with reduced stress, improved mood, enhanced resilience, and long-term identity development (Brymer et al., 2019; Brymer & Schweitzer, 2024; Frumkin et al., 2017; Hartig et al., 2014; Mitten & Brymer, 2022; Sharma-Brymer et al., 2025a; Wyles et al., 2017). Outdoor experiences have been found to support cognitive and social growth, as well as therapeutic outcomes such as emotional regulation, trauma recovery, and social inclusion (Bratman et al., 2012; Down et al., 2023; Depledge & Bird, 2009; Ewert et al., 2021).

However, research about nature-related health and well-being often emphasizes structured facilitation, individualistic growth, and measurable outcomes. Less attention has been paid to how healing unfolds through place-based, relational, and culturally embedded outdoor practices, particularly in postcolonial contexts like India, where Indigenous ecological knowledge, spiritual philosophies, and daily interactions with nature have long informed health and well-being (Mitra et al., 2024; Sharma-Brymer, 2022; Shiva, 2004). The following review synthesizes three core thematic strands that provide a theoretical foundation for understanding the therapeutic benefits of outdoor experiences in the Indian context.

#### 1.1.1. Nature as Emotional Regulator and Reflective Space

Natural environments have been found to act as co-regulators of emotional and physiological states. These processes may be especially significant for individuals experiencing grief, trauma, or burnout. For example, attention restoration theory (Kaplan & Kaplan, 1989) and stress reduction theory (Ulrich, 1984) suggest that natural settings gently engage attention and enable recovery from cognitive fatigue and emotional distress. The somatic experiencing framework (Haines, 2019; Levine et al., 2018) emphasizes the importance of sensory engagement and rhythmic environments in trauma recovery, supporting nature’s role in facilitating non-verbal emotional processing. In addition, ecopsychology (Roszak, 2001; Roszak et al., 1995) frames the emotional relationship with nature as reciprocal (Davis & Canty, 2013; Roze des Ordons & Hill, 2025). This perspective is reinforced by research showing that even brief exposure to nature can reduce anxiety and facilitate emotional clarity (Brymer & Schweitzer, 2017; Keniger et al., 2013; Richardson & McEwan, 2018). In India, the emotional and symbolic significance of sacred natural landscapes and pilgrimage practices historically entail ecological respect and present a unique culturally embedded model of healing (Kumar & Ginwala, 2021; Mazumdar & Mazumdar, 2004; Mitra et al., 2024; Sharma-Brymer, 2018, 2022; Sharma-Brymer et al., 2025b). For example, pilgrimage to holy shrines in rural and remote places in India is a common practice for Indian people. Pilgrims experience spirituality blended with respectful connection to nature. Non-pilgrims can also have adventurous and healing experiences resulting in body-mind-spirit transformation.

#### 1.1.2. Therapeutic Benefits of Human–Nature Relationships

Place attachment theory highlights the psychological importance of specific geographic familiarity in fostering emotional resilience, identity continuity, and a sense of belonging (Luong, 2025; Scannell & Gifford, 2010). Bioregional education builds on this by advocating for educational and therapeutic practices rooted in local ecological knowledge (Meadows, 1996; Sobel, 2004). Indigenous knowledge systems further expand this framework, emphasizing reciprocal relationships between humans and land, and viewing nature as both teacher and kin (Cajete, 1994; Kimmerer, 2013; Roze des Ordons & Hill, 2025; Sharma-Brymer et al., 2024; Simpson, 2014). Mazumdar and Mazumdar (2004) emphasize that sacred places such as rivers, forests, or pilgrimage routes carry emotional and symbolic weight that deepens the places’ therapeutic potential. Participants in such settings often report spontaneous reflection, spiritual insight, or cathartic release (Vijayakumar & Srivastava, 2024). In India, these principles are reflected in ancestral relationships with place, where forests, rivers, and mountains hold ecological, sociocultural and emotional memory (Ginwala et al., in press; Kumar & Ginwala, 2021; Sharma-Brymer, 2022; Shiva, 2016). Healing, in this context, emerges through a return to known ecosystems. Outdoor environments carry stories from religious and folktale contexts, offering specific place-based relationality between people and place. They are intergenerational lifeworlds that proffer special meanings to people who belong to the place.

#### 1.1.3. Decolonial, Bioregional, and Cultural Healing

The literature points to the damage caused by the erasure of traditional outdoor practices due to colonization. Scholars argue that colonial schooling systems displaced Indigenous pedagogies that had integrated embodied learning, human–nature relationships, and intergenerational knowledge (Battiste, 2013; Simpson, 2014; Smith, 1999). This displacement has contributed to cultural loss, as well as to a fragmentation of mental and ecological health. Reclaiming practices, such as forest bathing, river rituals, or seasonal awareness, has been shown to foster nurturing ecological identities, emotional stability, and ethical responsibility (Deloria & Wildcat, 2001; Jones & Segal, 2018; Kimmerer, 2013; Tuck et al., 2014). When doing so, outdoor experiences can become a form of cultural restoration, reconnecting learners with nature, culture, and relational ways of knowing. Programs that incorporate local stories, rituals, and ecological knowledge move beyond adventure or skill-building toward a pedagogy of healing.

Outdoor experiences that challenge physical, emotional, or social boundaries can lead to profound transformations in confidence and relational healing. Transformative learning theory (Caston, 2014; Mezirow, 2009) and experiential education (Dewey, 1938; Warren et al., 2008) emphasize the importance of reflection in creating psychological shifts. The community psychology literature reinforces that belonging is a key determinant of mental health (McMillan & Chavis, 1986; Roze des Ordons & Hill, 2025). Outdoor programs that foster inclusive, emotionally safe environments contribute to mental health by allowing participants to be seen, supported, and respected (Mitten, 1996, 2017).

Notably, many of these shifts occur outside formal instruction, emerging from shared silence, group member relationships, or unspoken understanding. The experience of awe, often invoked in sacred natural landscapes, has similarly been linked to emotional healing (Caston, 2014; Shiota et al., 2007). Brymer and Schweitzer (2017) suggested that profound therapeutic moments in nature arise from embodied connection. This echoes narratives from India, where transformation is often described as happening in the in-between moments—in the walk between activities, in the quiet reflection, or in the presence of caring others (Mitra, 2022; Sharma-Brymer, 2022). These unstructured transformative experiences in outdoor experiences support positive mental health outcomes. Drawing on these intersecting frameworks, this study investigates how outdoor practitioners in India perceive and promote healing and behavioral well-being through being outdoors and through their work.

## 2. Methodology

This study used an in-depth interview research design rooted in interpretivist and constructivist traditions, aiming to understand how outdoor practitioners in India perceive and articulate the therapeutic dimensions of outdoor experiences (Lincoln & Guba, 1985). This research involved 24 Indian practitioners with extensive experience in outdoor adventure education, adventure tourism, environmental conservation, and nature-based therapeutic work. Practitioners interviewed included outdoor educators, program facilitators, environmentalists, nature therapists, mountaineers, adventure leaders, and organizational founders. Many are recognized as founders or thought leaders in their respective fields. The table in Appendix A displays details about the study participants, including their names (see Section 2.3 Ethical Considerations), age, area of practice, and years in practice.

Sampling was purposive and snowball-based, beginning with established contacts in outdoor fields and expanding through referrals (Patton, 2014). This approach helped ensure that the interviewees possessed rich experiential knowledge and could offer diverse, contextually grounded perspectives on healing in outdoor settings. A range of genders, geographies, roles, and affiliations was intentionally included to allow for nuanced insights into India’s evolving outdoor landscape.

### 2.1. Data Collection

Data were gathered through semi-structured, in-depth interviews conducted between 2020 and 2022. Each interview lasted approximately 60 to 90 min and was conducted via Zoom, an online platform. The interviews, conducted in English, were audio-recorded with the participants’ consent and transcribed verbatim. A semi-structured interview guide was used to elicit narratives around the interviewee’s outdoor experiences. Therapeutic qualities of outdoor experiences, cultural practices, and inclusive program design surfaced as prominent themes during these semi-structured interviews. The researchers then probed further for clarity. The interviews were intentionally conversational in style, allowing participants to shape the flow and depth of their responses. Several interviewees, particularly those with therapeutic or cultural backgrounds, shared personal stories, traditional rituals, and embodied memories as part of their reflections.

### 2.2. Data Analysis

The thematic analysis followed a systematic and iterative process. Initial manual coding was performed on all 24 interview transcripts by the first author, highlighting key phrases, repeated concepts, and emotional markers. Coding and theme identification were conducted inductively, with codes and categories emerging organically from interviewee narratives as outlined by Braun and Clarke (2006). The initial coding looked at origins of outdoor practices in India.

For the present study, the co-authors reviewed the initial categories against the original transcripts to cross-check interpretations and ensure consistency. We undertook a framework-informed, deductive synthesis to organize and name the themes using an iterative process of looking at the data and the literature. Through collaborative discussions, the authors refined and condensed initial categories into three core themes: (a) nature as emotional regulation and reflective space, (b) therapeutic benefits of human–nature relationships, and (c) decolonial, bioregional, and cultural healing. When subthemes appeared to fit more than one framework, we kept multiple tags during synthesis and resolved naming through discussion to consensus among the co-authors. The deductive step was used to situate the inductive results for this study and to make our theoretical connections explicit. It did not replace or collapse the data-driven categories. Final theme determination continued to be driven by the inductive patterns.

Our approach ensured that the final thematic categories were both grounded in the data and informed by relevant scholarship. The themes reflect interviewees’ diverse perspectives and experiences, with representative quotations selected to illustrate how these themes emerged organically from interviewees’ narratives. We prioritized Indigenous and decolonial lenses to avoid reinscribing colonial logics, and we retained cases that resisted alignment; these are reflected in the Findings section.

### 2.3. Ethical Considerations

This study received ethical approval from the Prescott College Institutional Review Board. Participants were provided with an informed consent form detailing this study’s aims, procedures, and their rights, including the right to withdraw at any time without consequences. Given the public visibility of the interviewed practitioners within the Indian outdoor education fields, special attention was paid to representation. While all the interviewees consented to be named in this study, the researchers remained sensitive to how the narratives were presented to avoid misrepresentation or harm to professional reputations or personal lives.

Ethical challenges arose in relation to power dynamics and potential emotional risks, especially when experiences of exclusion, personal trauma, or systemic marginalization were discussed. The authors maintained a reflexive stance regarding their positionalities, given their diverse cultural and academic backgrounds—two authors from India and two from the USA, who range in age from 42 years old to over 70 and have over 80 combined years as practitioners and over 50 combined years as academicians. This diversity enriched interpretations and ensured cultural sensitivity. Additionally, the research team exercised caution when representing narratives, consciously respecting cultural contexts, personal sensitivities, and professional reputations. Quotes were carefully selected and contextualized to authentically reflect intended meanings and to avoid oversimplification.

## 3. Findings

Indian practitioners in this study described outdoor experiences as transcending recreation or performance—as deeply integrated into relational, emotional, and cultural processes that foster healing and behavior transformation. Following inductive coding and a framework-informed synthesis, we organized the data into three themes—(a) how nature acted as a space of emotional regulation and introspection, (b) how human–nature relationships held historical and spiritual significance, and (c) how place-based practices fostered critical resistance to dominant frameworks while affirming belonging and positive identity development. The findings are presented with discussion; practitioner insights are integrated with theoretical literature to emphasize how these lived experiences reflect broader psychological and sociocultural dynamics. Each theme is supported by verbatim quotes to illustrate how meaning is constructed. The findings, presented and discussed under the three core themes, represent the complexity and coherence of the data in relation to Indian outdoor practitioners’ relationship to nature as they practice their craft and participate in outdoor activities.

### 3.1. Nature as Emotional Regulation and Reflective Space

Across narratives, nature was consistently described as a catalyst for self-regulation and introspection, which can lead to transformation. Practitioners emphasized how unstructured time in natural spaces fostered inner calm, resilience, and mental renewal, often providing a refuge from urban life or personal distress. One interviewee, Ravi Kumar, recalled how time spent in nature offered grounding: “I always felt calm, composed, it gave me some sort of a deeper satisfaction when I was in the outdoors versus being in the classroom in the school,” he continued, “In all that greenery and the wilderness, I felt really small. That was a turning point for me.” In Indian contexts, this effect of nature is often amplified by the cultural reverence for sacred landscapes and symbolic interactions with the environment.

The transformational power of a 7-day hike was profound in that it shifted a practitioners’ outlook on himself and inspired him to create similar opportunities for youth, particularly those grappling with challenging behaviors. He reflected on how the outdoors provided a space for personal growth and emotional transformation, moving from a self-described place of being ‘mean’ and ‘a bully’ to one of humility and care for others. The therapeutic qualities of nature were often associated with its capacity to hold silence, support grief processing, and provide space for non-verbal emotional release. This echoes principles of somatic psychology, where sensory grounding in nature through breath, touch, or rhythm enables deep regulation of stress and trauma (Haines, 2019). Indian practitioners often framed this as a return to the self, facilitated by trees, rivers, and wind. Similarly, Sarabjit Singh Wallia described the psychological strength that nature helped build:

When stuff happens, you don’t give up. That’s it. It’s how it is. In mountaineering you know this: that failure is a very regular part of your day. Don’t get bogged down by it. So, it makes you emotionally stronger; it makes you mentally stronger.

These reflections highlight how outdoor experiences for Indian practitioners in India function as affective environments that support emotional regulation, psychological strengthening, and reflection.

Beyond individual healing, practitioners interviewed noted how spending time in nature enabled clarity of thought. Silence was described as an active presence that allowed emotions to surface and settle. Practitioners also recognized nature’s capacity to transform emotional energy into behavioral change, whether through adventurous or contemplative activities. As Manjul Prateeti explained, “It [behavioral change] can be through traditional adventure activities. It can be sitting underneath the tree and reflecting and having some thought process that makes you start to learn something about yourself.” This duality of active and passive engagement highlights the diverse ways outdoor spaces foster self-awareness and personal growth. These participant reflections align with theoretical frameworks such as attention restoration theory (Kaplan & Kaplan, 1989) and stress reduction theory (Ulrich, 1984), which suggest that natural settings gently engage attention and facilitate emotional recovery. Furthermore, the somatic experiencing framework (Haines, 2019) emphasizes the role of sensory grounding in nature for deep stress and trauma regulation. Ecopsychological and emotional perspectives (Roszak, 2001; Roze des Ordons & Hill, 2025) similarly frame nature as relational, supporting emotional complexity and prosocial behaviors. Their reflections affirm prior research suggesting that nature immersion supports prosocial behavior, increased emotional openness, and cooperative group dynamics (Brymer & Schweitzer, 2017). In Indian settings, this shift was often framed as spiritual, which allows for a reconnection with something greater than the self, mediated by landscape.

### 3.2. Therapeutic Benefits of Human–Nature Relationships

The practitioners interviewed understood human–nature relationships within the contexts of India’s long-standing spiritual and ecological traditions. They emphasized healing as something embedded within relationships—relationships with land, with community, and with one’s own cultural memory. Indian people’s outdoor experiences were holistic, supporting healing through emotional, cultural, and relational threads. These understandings align with Indigenous and decolonial paradigms that view well-being as rooted in reciprocity and relationality (Cajete, 1994; Kimmerer, 2013; Roze des Ordons & Hill, 2025). Interviewees often drew from spiritual geographies, such as pilgrimage sites, rural landscapes, and ancestral traditions, that facilitated reconnection with values, identity, and community. Nomito Kamdar shared the following:

People went for pilgrimage. We call it trekking today but when people went for pilgrimages, they walked up the hill to reach the temple and then when they reached the temple, they felt that they had met God, or they felt peace. Is that not why we go to the wilderness also?

This understanding contests the dominant narrative of nature engagement as a modern leisure activity, instead highlighting its continuity with sacred movement and reflective travel. Similarly, Ravi Kumar highlighted India’s historical connections to nature:

In the past, part and parcel of India’s culture and life was schooling in the wilderness. Most young people were sent away into the forest, to live with the monks and learn for themselves with the guidance of the monks which is nothing but experiential education. And it was intense. And when they were ready 16–17 years old is when they would graduate and come back to the society. So, the idea was that if you experience hardship, dealing with adversities and for an extended period of time, it would give you life skills to become a better human being.

Through such reflections, study interviewees suggested that outdoor experiences were embedded within a civilizational ethos where emotional growth, moral development, and ecological awareness were practiced as part of daily living. These perspectives echo that healing does not separate itself from environmental and spiritual interconnectedness (Simpson, 2014).

For many practitioners, healing was catalyzed not just by the beauty or quiet of nature but by the cultural resonance of specific sites—rivers that held generational memory, forests revered by local communities, or mountain passes associated with myths and metaphors. Deepti Bhat shared how guiding children through mangrove ecosystems evoked emotional and sensory responses:

One of my favorite spaces till now has been the mushy mangroves. I love taking kids on these mangrove walks because I love to see their expressions. It takes so much time for them to just get used to the land and the walk. And I love that space because it brings a lot of emotions out from the students as well.

In this context, the outdoor space was not neutral. It was embedded with meaning, history, and potential that foster ecological awareness and emotional connection. Divyanshu Ganatra emphasized this reciprocal space-making: “When you come and play in the outdoors, it helps build empathy, it helps dialogue, it fosters friendships.” These insights reinforce that therapeutic value is not a modern import but an embedded element of Indian ways of life. Sacred journeys, rural livings, and spiritual landscapes shaped early exposure to the outdoors, making healing in nature a culturally resonant practice rooted in intergenerational knowledge and ecological identity.

Importantly, the human–nature relationship in these narratives was not unidirectional. Practitioners did not speak of “using” nature for healing but of relating to it in and outside of their professional work, being humbled by it, and advocating for its care. This ethical sensibility appeared central to their understanding of mental health as a personal state as well as a practice of living well in relation to others, including non-human others. By reconnecting contemporary practice with ancestral ecologies, interviewees challenged both Western clinical framings of therapy and neoliberal environmental narratives that commodify nature. Instead, they offered a vision of therapeutic engagement grounded in cultural memory, humility, and respect. These human–nature relationships supported emotional integration and cultivated behaviors such as empathy, gratitude, and collective responsibility. Participants’ narratives in this study reveal that healing arises from the ethical, historical, and relational layers that shape their relationships with natural environments. Such insights urge scholars and practitioners to expand their conceptualizations of outdoor therapy—to include land as a sentient force shaping the therapeutic space, traditions as a compass, and community as a resource for sustainable well-being.

### 3.3. Decolonial, Bioregional and Cultural Healing

The final theme centers on the decolonial and bioregional dimensions of healing, with practitioners emphasizing the need to reclaim traditional relationships with land, resist elitist outdoor narratives, and center inclusive and culturally grounded practices. As Deepti Bhat reflected,

I felt that outdoors has always been a part of people’s life in India. If you go to any rural space, people are always outdoors, they learn from outdoors, the learning is always there. So, it has been given a name, outdoor education, maybe I also say it has a colonial influence in a way that it has come in as outdoor education in our lives and how we teach kids using outdoor education. But in my opinion, if you talk about history of outdoor education, it has always been there in the lives of the people.

This observation challenges the assumption that outdoor education is a Western import and situates it as an Indigenous, historically embedded practice in Indian cultural life. Like Deepti Bhat, several practitioners argued that most outdoor fields, such as outdoor adventure education and adventure tourism in India, have long been influenced by Western adventure and leadership models that often ignore Indigenous ecological wisdom and local lifeways (Mitten, 2021). Indian practitioners interviewed advocated for a practice of outdoor engagement that is grounded in local bioregions, ancestral knowledge, and cultural rituals. Several critiqued the tendency to frame outdoor engagement as a Western import. Anindya Mukherjee spoke about Indian people’s long custom of respectful relationships with nature: “Traditionally, we Indians never looked at the mountains as something to conquer. We look [at them] as a pilgrimage or a place to go for meditation or worshipping.” His perspective reveals a historical ontology where nature was beyond an object of conquest but instead a site of respect, reflection, and humility. This reframing also underscores the importance of dismantling colonial narratives embedded in outdoor narratives.

The importance of re-centering Indian frameworks was echoed by Ajay Rastogi: “We consume outdoors for education, for adventure, or for entertainment. There is a fundamental difference in being in it [nature] and not just considering that it is a utility, in way of consuming, like transactional.” For him and others, ecological healing is inseparable from a shift in worldview—from one that objectifies nature to one that honors nature as a relational entity. This aligns with global scholarship on bioregional education and Indigenous land pedagogies (Battiste, 2013; Cajete, 1994; Simpson, 2014; Smith, 1999; Tuck et al., 2014), which promote local ecological immersion and cultural continuity as foundations for psychological and social well-being.

Tanya Ginwala, an outdoor therapist, emphasized the need to be cautious with romanticized narratives of nature, counseling against the colonial stereotype of associating the outdoors as a safe place for all. She said, “I don’t come in with this assumption that everybody loves being outside. I think everyone can grow to love it because it is amazing. But I don’t come in with this ‘how can you not love to be outside?’” Her practice emphasized permission, pacing, and honoring contradiction, particularly for participants whose relationships to land had been shaped by caste, gender, or trauma. In essence, she invites a non-dominant perspective of outdoor as therapeutic intervention and in practice operationalizes how to be inclusive when introducing or reintroducing people to natural environments. Practitioners stressed that inclusive practices must go beyond colonial rhetoric. They shared how they adapted program designs to local languages, oral storytelling traditions, and culturally significant metaphors. This theme reveals that bioregional and cultural healing requires a decolonial shift in how outdoor experiences are conceptualized and facilitated. Rather than exporting frameworks from the Eurocentric practices, Indian practitioners are drawing on their own cultural legacies to design experiences that are emotionally safe, culturally inclusive, and ecologically grounded. Healing for them emerges from relational reweaving with land, with ancestors, and with each other. These practices support mental health and ethical, sustainable behaviors that honor India’s diverse ecological and spiritual traditions.

## 4. Discussion

### 4.1. Contributions to the Literature

This study contributes to the growing body of literature on the therapeutic benefits of nature by grounding it in a specific cultural and ecological context. While global research increasingly acknowledges the mental health impacts of outdoor experiences, few studies explore these dynamics in postcolonial, non-Western settings. By centering Indian practitioners’ voices, this research offers a decolonial perspective that broadens our understanding of how healing in nature is conceptualized and practiced. It underscores the importance of integrating cultural epistemologies and historical consciousness into outdoor education and therapy frameworks. This work advances theoretical conversations in ecopsychology, experiential education, and Indigenous pedagogy while highlighting culturally rooted expressions of emotional well-being and behavioral transformation.

### 4.2. Practical Implications

The practical implications offer ideas for designing culturally responsive, emotionally safe, and ecologically grounded nature-based practices that honor Indian traditions and historic relationships with nature. Many outdoor practitioners in India already understand the importance of working with nature (and not ascribing utility to it) as the primary reason for engaging with nature. Ginwala et al. (in press) specifically focuses on the therapeutic value of time in nature for healing in human relationships, including grief, and in strengthening healthy relationships with nature to encourage continuing reciprocal relationships with nature for individuals, social groups, and families.

Practitioners and others are encouraged to relate outdoor activities to the outdoor traditions associated with various bioregions and with India. This includes pilgrimages to important sacred sites, including certain mountain tops, and relating these journeys to spiritual traditions. Highlighting the spiritual aspect of outdoor journeys honors traditions and helps inclusion of the many reasons to engage in relationships with nature.

Practitioners can maintain discussions among themselves and with participants about ways to further decolonize their outdoor experiences. Indian practitioners are encouraged to continue to be part of the international conversations, highlighting Indigenous traditions and decoupling these from elitist, Western-oriented frameworks of outdoor practices that can dominate training and practices.

### 4.3. Limitations and Future Research

This study is based on a qualitative analysis of 24 semi-structured interviews with experienced outdoor practitioners in India. While this approach provided rich, nuanced insights into culturally embedded therapeutic practices, it also limited the generalizability of the findings to broader populations or contexts. Most study participants were fluent in English and identified via professional networks, potentially overrepresenting urban, educated, or leadership voices in the field. Additionally, participants’ reflections were shaped by their own roles as facilitators, which may differ from the experiences of outdoor program participants or community members themselves.

Language and cultural translation also posed additional limitations. All interviews were conducted in English, which was not the first language for most participants. Though efforts were made to ensure clarity and allow for conversational flow, certain expressions or cultural idioms may have been constrained by linguistic limitations. Future research should explore multilingual approaches and regional languages to capture deeper layers of meaning and cultural nuance, particularly when working across diverse socio-economic and geographic backgrounds.

Further studies are needed to explore the perspectives of outdoor participants, especially ones from marginalized or underrepresented communities such as tribal groups, rural youth, and gender-diverse individuals. Mixed-methods research that combines qualitative inquiry with quantitative assessments could help examine behavioral outcomes more systematically. Longitudinal studies that track changes in emotional regulation, pro-social behaviors, and ecological attitudes over time may offer robust insights into the sustained impact of culturally grounded outdoor education. Exploring how these practices are being adapted for urban or digitally mediated environments may also be a valuable direction for future scholarship.

## 5. Conclusions

The researchers in this study invite Indian practitioners to continue to reflect on their practices to ensure they promote practices to support positive and healthy relationships with nature that honor and encourage traditional Indian ecological values. Interviewees in this research study described the outdoors and nature as relational, somatic, and culturally rooted in ways that help participants—both practitioners and the people they facilitate—develop more emotional regulation, identity integration, and social inclusion. Many of the outdoor practices used facilitate healing through solitude and silence, as well as ancestral connection, exposure to sacred landscapes, inclusive group dynamics, and the restoration of embodied, cultural knowledge. As global attention to mental health and nature-based interventions continues to grow, it is vital that such conversations include perspectives that have often been marginalized. By foregrounding Indigenous, postcolonial, and bioregional understandings, this study offers a pathway toward more equitable and resonant practices in outdoor mental health and education worldwide.

## Data Availability

The data presented in this study are available on request from the corresponding author due to privacy and ethical restrictions.

## Appendix A

**Table A1:** Research participants.

Name	Age at the Time of Interview (2022)	Gender	Practice Area	Practicing Since
Ajay Rastogi	57	Man	Outdoor education, nature conservation	2008
Akshay Shah	53	Man	Outdoor education, wilderness medicine	1991
Anindya Mukherjee	51	Man	Mountaineering, mountain literature	1994
Arjun Majumdar	50	Man	Outdoor recreation and education	2008
Deepti Bhat	32	Woman	Conservation and environmental education	2011
Divyanshu Ganatra	45	Man	Social justice, inclusivity in outdoor education	2016
Gautam Dutta	62	Man	Outdoor education	1990
K Krishan Kutty	62	Man	Outdoor education	1987
Kapil Bhalla	66	Man	Outdoor education	2012
Madhu Sudan G	42	Man	Outdoor education	1999
Manjul Prateeti	32	Woman	Outdoor education	2010
Nomito Kamdar	55	Woman	Social justice, inclusivity in outdoor education	1979
Pavane Mann	65	Woman	Outdoor education	1990
Ravi Kumar	55	Man	Outdoor education, wilderness medicine	1994
Sarabjit Singh Wallia	43	Man	Outdoor education	2000
Shantanu Pandit	59	Man	Outdoor education, wilderness medicine	1989
Sohan Pavuluri	47	Man	Outdoor education	2015
Sudhir Moharir	65	Man	Outdoor education	2000
Tanya Ginwala	31	Woman	Outdoor therapy	2015
Tarun Chandna	55	Man	Outdoor education	1996
Usha Ramaiah	81	Woman	Outdoor education	1966
Vasant Limaye	67	Man	Facilitation, outdoor education	1978
Vinay Sirsi	50	Man	Facilitation, outdoor education, wilderness medicine	1997
Vishwas Parchure	65	Man	Facilitation, outdoor education	1989
